# *LPCAT1* overexpression promotes the progression of hepatocellular carcinoma

**DOI:** 10.1186/s12935-021-02130-4

**Published:** 2021-08-21

**Authors:** Rong-Quan He, Jian-Di Li, Xiu-Fang Du, Yi-Wu Dang, Lin-Jie Yang, Zhi-Guang Huang, Li-Min Liu, Liu-Feng Liao, Hong Yang, Gang Chen

**Affiliations:** 1 Department of Oncology, First Affiliated Hospital of Guangxi Medical University, No. 6 Shuangyong Rd, Guangxi Zhuang Autonomous Region, Nanning, 530021 People’s Republic of China; 2 Department of Pathology, First Affiliated Hospital of Guangxi Medical University, No. 6 Shuangyong Rd, Guangxi Zhuang Autonomous Region, Nanning, 530021 People’s Republic of China; 3 Department of Toxicology, College of Pharmacy, Guangxi Medical University, No. 22 Shuangyong Rd, Guangxi Zhuang Autonomous Region, Nanning, 530021 People’s Republic of China; 4 Department of Pharmacy, Guangxi Medical University Cancer Hospital, No. 71 Hedi Rd, Guangxi Zhuang Autonomous Region, Nanning, 530021 People’s Republic of China; 5 The Ultrasonics Division of Radiology Department, The First Affiliated Hospital of Guangxi Medical University, No. 6. Shuangyong Rd, Guangxi Zhuang Autonomous Region, Nanning, 530021 People’s Republic of China

**Keywords:** Hepatocellular carcinoma, Lysophosphatidylcholine acyltransferase 1, Functional experiment, Molecular mechanism, Clinical applications

## Abstract

**Background:**

Hepatocellular carcinoma (HCC) remains one of the most common malignant neoplasms. Lysophosphatidylcholine acyltransferase 1 (*LPCAT1*) plays a key role in the lipid remodelling and is correlated with various neoplasms. Nonetheless, the biological functions and molecular mechanisms of *LPCAT1* underlying HCC remain obscure.

**Methods:**

In the present study, we investigated the role of *LPCAT1* in the progression of HCC. In-house RT-qPCR, tissue microarrays, and immunohistochemistry were performed to detect the expression levels and the clinical value of *LPCAT1* in HCC. External datasets were downloaded to confirm the results. Proliferation, migration, invasiveness, cell cycle, and apoptosis assays were conducted to reveal the biological effects *LPCAT1* has on SMMC-7721 and Huh7 cells. HCC differentially expressed genes and *LPCAT1* co-expressed genes were identified to explore the molecular mechanisms underlying HCC progression.

**Results:**

*LPCAT1* showed upregulated expression in 3715 HCC specimens as opposed to 3105 non-tumour specimens. Additionally, *LPCAT1* might be an independent prognostic factor for HCC. *LPCAT1*-knockout hampered cellular proliferation, migration, and metastasis in SMMC-7721 and Huh7 cells. More importantly, the cell cycle and chemical carcinogenesis were the two most enriched signalling pathways.

**Conclusions:**

The present study demonstrated that increased *LPCAT1* correlated with poor prognosis in HCC patients and fuelled HCC progression by promoting cellular growth, migration, and metastasis.

**Supplementary Information:**

The online version contains supplementary material available at 10.1186/s12935-021-02130-4.

## Background

Hepatocellular carcinoma (HCC), characterized by high molecular heterogeneity, serves as the predominant subtype of liver cancer [1, 2]. HCC is the third most frequent cause of cancer deaths worldwide, and ranks fifth and seventh in male and female malignancy [3], respectively. According to American Cancer Statistics 2021, there will be approximately 40,000 new cancer cases and 30,000 deaths per year, with a 5-year relative survival rate of only 20% [3]. HCC is a complex and multistage disease that may be explained by genetic and epigenetic changes [4–8]. Despite the development of surgery and combined treatment, as well as progress in early diagnosis and interventional therapy, most patients are still diagnosed in the advanced stage of HCC with a low 5-year survival rate and poor prognosis [9–11]. Therefore, clarifying the pathogenic mechanisms of HCC is essential for establishing novel potential therapeutic strategies and improving the prognosis of advanced stage HCC patients.

As a basic material of life, phospholipids play an important role in maintaining normal cellular structures, homeostasis, and fundamental cellular biological functions [12]. However, abnormal lipid metabolism disturbs cellular activities and drives cancer formation by affecting cell differentiation, proliferation, and apoptosis [13]. Lysophosphatidylcholine acyltransferase 1 (LPCAT1), encoded by *LPCAT1*, is a phospholipid biosynthesis/remodelling enzyme that plays a key role in the lipid remodelling pathway referred to as Lands’ cycle [14, 15]. LPCAT1 participates in phospholipid metabolism by catalysing the conversion from lysophosphatidylcholine to phosphatidylcholine and has been demonstrated to be correlated with various neoplasms, including hepatoma, acute myeloid leukaemia [13], breast cancer [16], clear cell renal cell carcinoma [17], colorectal cancer [18], esophageal cancer, lung adenocarcinoma [19], oral squamous cell carcinoma [20], and prostate cancer [21]. Thus far, only one study, limited in sample size, demonstrates that *LPCAT1* is upregulated in 37 HCC tissue samples [22]. During the process of tumour initiation and progression, increased *LPCAT1* promotes the synthesis and remodelling of lipids in the lipid-dependent membrane structures of rapidly proliferating cancer cells [16]. Additionally, *LPCAT1* induces metastasis of cancer through complicated mechanisms, such as the lipid metabolism pathway and PI3K/ AKT/ MYC pathway [23]. Moreover, the alteration of the phospholipid component regulated by *LPCAT1* promotes cell proliferation and membrane fluidity, thus causing the occurrence and development of clear cell renal cell carcinoma [17]. Collectively, such findings indicate the vital roles *LPCAT1* play in tumour onset and progression. Nonetheless, the exact biological functions and precise molecular mechanisms of *LPCAT1* underlying HCC remain obscure. More experiments must be conducted to determine the key roles of *LPCAT1* in HCC and to design novel therapeutic targets.

In this study, the authors aimed to verify the expression of *LPCAT1* in large numbers of clinical HCC tissue samples and to explore the biological functions of *LPCAT1* in HCC cells. Furthermore, the potential molecular mechanisms and therapeutics of *LPCAT1* underlying HCC were probed.

## Methods

### HCC-derived cell lines and tissue samples

HCC-derived SMMC-7721 and Huh7 cell lines were obtained from the Cell Bank of the Chinese Academy of Sciences, Shanghai, People’s Republic of China [24]. The cellular identity was confirmed by short tandem repeat profiles. All cells were cultured in Dulbecco’s modified Eagle’s medium (DMEM) containing 10% fetal bovine serum (FBS) (Sigma), as formerly reported [25].

To detect the mRNA expression level of *LPCAT1*, 204 HCC tissue specimens and 204 non-tumour hepatic tissue specimens were obtained from the Affiliated Cancer Hospital of Guangxi Medical University, Nanning, People’s Republic of China. The clinicopathological parameters and follow-up information of each patient were recorded. Informed consent was provided. To determine the protein expression level of *LPCAT1* in HCC, a total of four tissue microarrays, containing LVC1505, LVC1531, LVC1601, and LVC1602, were purchased from Fanpu Biotech, Inc. (http://www.fanpu.com), Guilin, People’s Republic of China. A total of 315 HCC tissue specimens and 195 non-HCC tissue specimens were prepared to perform immunohistochemistry (IHC) staining. The inclusion criteria for patients in this study are as follows, (I) the pathological diagnosis should be primary HCC; (II) there should be sufficient tissue specimens for RNA extraction or IHC. To avoid confounding effect, HCC patient with intrahepatic cholangiocarcinoma was excluded in this study. The present research was authorised by the ethics committee of the First Affiliated Hospital of Guangxi Medical University.

### In-house real-time reverse transcription-polymerase chain reaction, tissue microarrays, and immunohistochemistry

Fresh HCC tissue specimens and adjacent normal hepatic tissue specimens were prepared to extract total RNA by employing the AxyPrep Multisource Total RNA Miniprep Kit (AP-MN-MS-RNA-250G, AXYGEN, China). RNA was reversely transcribed into cDNA using the ‘Takara PrimeScript RT reagent’ kit (Takara, Nanning, People’s Republic of China, as we previously reported [26]. Beta-actin (*ACTB*) was used as the internal reference gene (forward sequence: “5′-CAGGCACCAGGGCGTGAT-3′”; reverse sequence: “5′-TAGCAACGTACATGGCTGGG-3′”) [27]. The primer of the target gene was designed on the mRNA sequence between the two gRNA binding sites (forward sequence: 5′-GTGACCATGACGATGTCCTCC-3′; reverse sequence: 5′-GTTCCCCAGATCGGGATGTC-3′). An algorithm of 2^−Δcq^ was used to determine the relative mRNA expression level of *LPCAT1* [28–30].

The protein expression status of *LPCAT1* in HCC was detected by conducting IHC, which was based on tissue microarrays. All the experimental operations concerning tissue microarrays and IHC staining follow the instructions of the manufacturer. The interpretation of staining intensity was performed by three expertized pathologists, according to the previously reported criteria [31].

### In-silico data mining of HCC RNA sequencing and gene chip datasets

The mRNA expression profiles of HCC submitted before October 2020 were queried and filtered from public biomedical databases, including Gene Expression Omnibus, ArrayExpress, The Cancer Genome Atlas, Sequence Read Archive, The Genotype-Tissue Expression, and scientific literature. The inclusion criteria were as follows: (I) the subjects included in the studies should be *Homo sapiens*; (II) the patients should be diagnosed with HCC; (III) the platform information should be provided to annotate each probe. The exclusion criteria were as follows: (I) the subjects were animals; (II) valid data related to *LPCAT1* expression was not presented; (III) duplicated data was reported; (IV) publications were in the form of case reports, reviews, and editorials. Three authors performed datasets screening independently, and an agreement was reached. A log_2_(x + 1) transformation was considered if the expression matrices had not been normalized. The included datasets were classified according to their affiliated sequencing agencies. Those datasets that belonged to the same sequencing agency were integrated into a larger expression matrix, and the inter-batch differences were removed by using Limma-Voom and sva packages in R v.3.6.1 software. The batch-removed expression matrices were prepared for gene analysis.

### In vitro cellular biological experiments

#### Construction of recombinant plasmids with the clustered regularly interspaced short palindromic repeats (CRISPR)/associated protein 9 (Cas9) system

We designed two gRNA sequences gRNA1: 5′-GGCGTCGAAGTAGGACGAGTG-3′ and gRNA2: 5′-GTCTGACCGGGACACGAACAC-3′. By synthesizing the oligo-DNAs of these gRNAs and annealing them, then cloning them into backbone vectors ‘pSpCas9(BB)-2A-Puro (PX459) V2.0’ (Plasmid #62988, addgene), two gRNA-expressing plasmids were formed [32].

#### Transfection with plasmids

HCC-derived cells (SMMC-7721 and Huh7) were stably transfected through lipo3000. After transfection for 24 h, the complete medium containing 2 μg/ml puromycin was screened for 48 h, and then the medium was transferred to the complete medium without puromycin to continue the culture. Transfected cells were used for further experiments.

#### Knockout effect verification

RT-qPCR was used to check the gene knockout efficiency. *ACTB* was used in combination as the internal reference gene. The primers of *ACTB* and the target gene were aforementioned in the “In-house real-time reverse transcription-polymerase chain reaction, tissue microarrays, and immunohistochemistry” section.

#### Cell viability

Proliferation assay was used to detect the effect of *LPCAT1* knockout on cell viability. Total 2 × 10^3^ transfected cells were seeded in each well of a 96-well plate. The cells were counted every individual day and lasted for five days. The numbers of cells between the experimental group and the blank control group were compared, and the cell growth curve was drawn.

#### Migration assay

Cell migration ability was measured using a transwell cell in a 24-well plate (corning, Costar 3422). A total of 100 μl medium (0.5 × 10^5^ cells) was added to the upper chamber, and 500 μl of medium was added to the lower chamber. The mediums in the upper and lower chambers were DMEM containing 5% FBS. After incubation at 37 °C for 24 h, the cells on the upper surface of the chamber were gently wiped with cotton swabs [20], washed twice with phosphate buffered solution (PBS), fixed in methanol for 30 min, dried and dyed in 0.1% crystal violet for 30 min, and washed with PBS to remove the excess dye solution. The dyeing solution was observed under a microscope after drying. A total of five fields per chamber were subjected for the migrated cell counting.

#### Invasiveness assay

Cell invasiveness was measured using a transwell cell in a 24-well plate (corning, Costar 3422). Matrigel (BD, 356234) was diluted with serum-free DMEM. The ratio of Matrigel to DMEM was 1:8. 60 μl of the diluted Matrigel was added to the chamber. After incubation at 37 °C for 1 h, the chamber was taken out and the liquid was aspirated before DMEM was used to wash it twice. A total of 100 μl of serum-free DMEM (0.5 × 10^5^ cells) was added into the chamber, and 500 μl of DMEM containing 5% FBS was added into the lower chamber and cultured at 37 °C for 24 h. A total of five fields per chamber were subjected for the invaded cell counting.

#### Cell cycle and apoptosis

The cell cycle distribution and apoptotic rate of the *LPCAT1*-knockout group and control blank group were detected using a flow cytometer in SMMC-7721 and Huh7 cells.

### Prospective molecular mechanisms of* LPCAT1* in HCC

HCC differential expression genes (DEGs, |log_2_FoldChange|> 1 and P < 0.05) were initially filtered from the batch-removed expression matrices by using the Limma package in R software v3.6.1. Standardized mean differences (SMD, |SMD|>0 and P < 0.05) were then quantitively calculated to identify DEGs, which were more reliable [33]. *LPCAT1* co-expressed genes (CEGs, Pearson Correlation Coefficient, |PCC|≥ 0.3 and P < 0.05) were filtered from the batch-removed expression matrices. Specifically, one gene with a PCC value ≥ 0.3 were defined as positive CEGs, while genes with a PCC value ≤ − 0.3 were defined as negative CEGs. DEGs and CEGs were intersected to analyse the clustered Gene Ontology (GO) terms, Disease Ontology (DO), reactome, and Kyoto Encyclopaedia of Genes and Genomes (KEGG) pathways by using the clusterprofiler package. The Search Tool for the Retrieval of Interacting Genes (STRING) database was used to analyse protein–protein interaction (PPI) [34]. Functional module analysis was conducted by using an algorithm of vertex-weighted MCODE in Cytoscape v3.6.1 software [35]. The hub gene in the PPI network was identified using Cytoscape. The genetic alterations and mutations of specific genes enriched in the first ranked KEGG pathways were analysed using the maftools package and cBioportal [36].

### Potential small molecule agents for HCC treatment

Although increasing novel cancer targets are reported, their practical implications are abrogated by poor druggability of such targets [37]. However, a connectivity map (Cmap) provides an opportunity to repurpose Food and Drug Administration-approved drugs according to specific gene signatures, which is a potential strategy for precision treatment of cancer [38–40]. By targeting at the gene regulatory network of the DEGs that were co-expressed with *LPCAT1* in HCC, several small molecule drugs for HCC treatment were forecasted.

### Data analysis

All the data were analysed under IBM Statistical Product and Service Solutions Statistics v19.0, STATA v12.0, and R v3.6.1 software. An independent samples t-test was used to compare the *LPCAT1* expression data in HCC tissues and non-tumour hepatic tissues. To combine SMD and hazard ratio (HR), a fixed-effect model was first considered when the I^2^ < 50%. A randomized-effect model should be used to handle the significant heterogeneity (I^2^ ≥ 50%). Sensitive analysis was adopted to detect the source of heterogeneity. Begg’s funnel plot was used to explore publication bias. The receiver operating characteristic curve (ROC) was plotted to determine the sensitivity and specificity. Summary receiver operating characteristic (SROC) curves were utilized to describe the area under the curve (AUC), pooled sensitivity, and pooled specificity. The likelihood ratio was used to determine the discriminatory ability of *LPCAT1* between HCC and non-HCC tissues. Kaplan–Meier analysis was performed to assess the prognostic value of *LPCAT1*. P < 0.05 signified significance.

## Results

### Increased LPCAT1 mRNA expression in HCC tissues

**Fig. 1:**
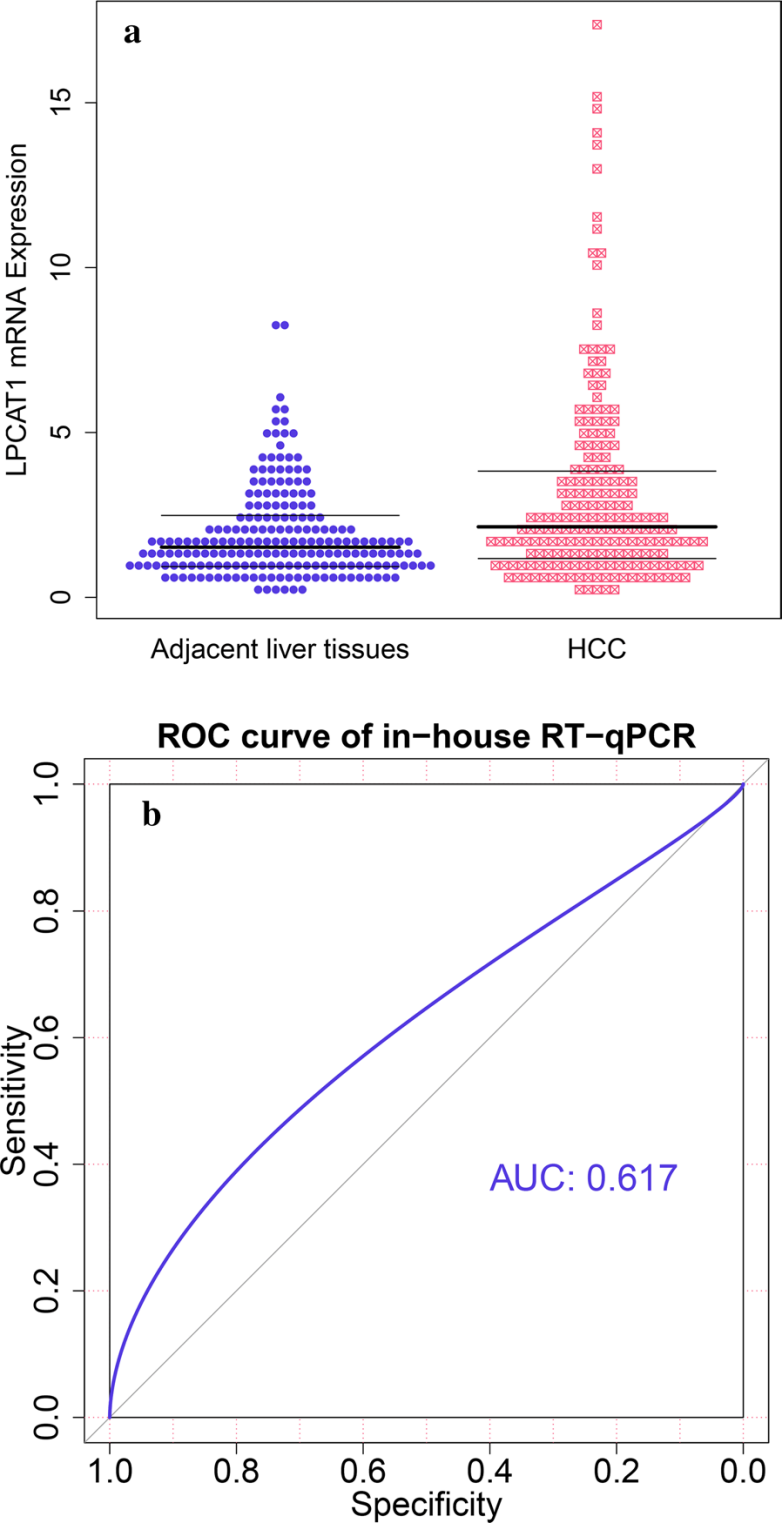
Upregulation of *LPCAT1* mRNA in hepatocellular carcinoma based on in-house real-time reverse transcription-polymerase chain reaction. **a** *LPCAT1* mRNA expression levels are markedly increased in hepatocellular carcinoma tissues. **b** Increased *LPCAT1* possessed a weak ability to discriminate between HCC and normal liver tissues. ****P < 0.0001

The result of in-house RT-qPCR indicated that *LPCTA1* mRNA was dominantly increased in 204 HCC tissue specimens as opposed to 204 non-tumour liver tissue specimens (Fig. 1a; Additional file 1: Table S1). Increased *LPCAT1*, however, possessed a weak ability to discriminate between HCC and normal liver tissues (Fig. 1b), which was due to a relatively small sample size. Eight out of ten external datasets confirmed the upregulated *LPCAT1* expression level (Additional file 2: Figure S1), where *LPCAT1* showed a moderate or strong ability to discriminate between HCC and normal liver tissues in four subsets. The basic information of such datasets was presented in Additional file 1: Table S1 and Additional file 3: Table S2. Collectively, a combined SMD value of 0.63 [0.35, 0.90] integrated in-house RT-qPCR results and external datasets and verified the increased mRNA expression levels of *LPCAT1* in 3715 HCC tissue samples in comparison with 3105 non-HCC tissue samples (Additional file 4: Figure S2a). A randomized-effects model was utilized to handle the significant heterogeneity (P < 0.01). The result of the sensitive analysis failed to identify a heterogeneity source (Additional file 4: Figure S2b). No publication bias was detected (Additional file 4: Figure S2c). The result of SROC indicated a moderate ability of *LPCAT1* in differentiating between HCC and non-HCC (AUC = 0.80 [0.77, 0.84]; sensitivity = 0.59 [0.42, 0.74]; specificity = 0.85 [0.74, 0.92]; combined diagnostic likelihood ratio positive = 3.98 [2.32, 6.82]; and combined diagnostic likelihood ratio negative = 0.48 [0.34, 0.69]) (Additional file 5: Figure S3a–c).

### Increased LPCAT1 protein expression level in HCC tissues

**Fig. 2:**
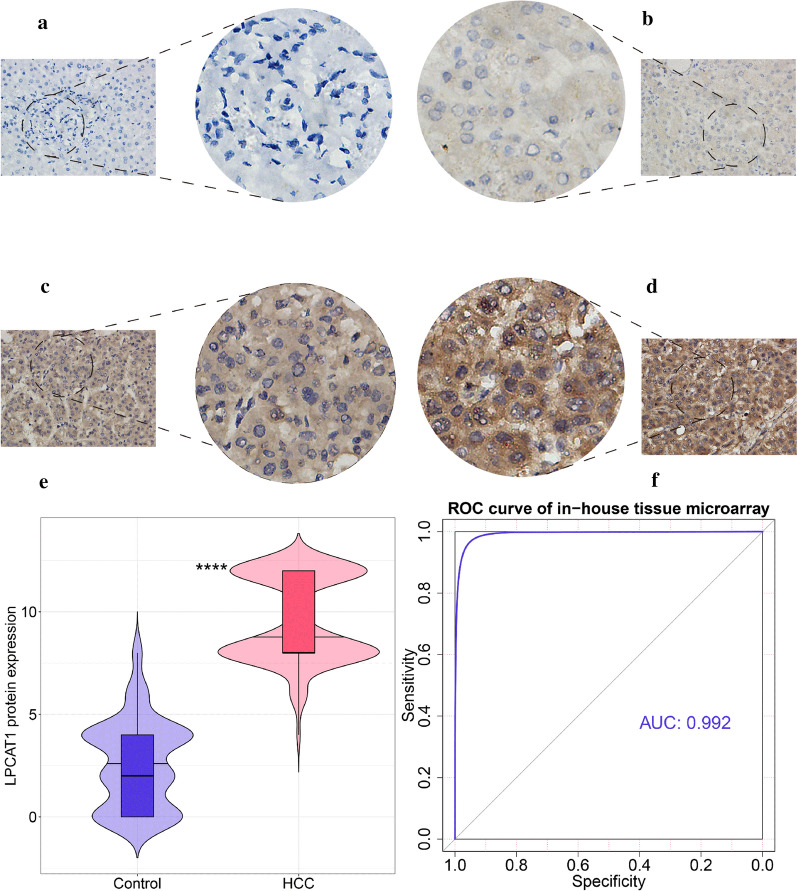
Upregulation of LPCAT1 protein in HCC based on immunohistochemistry. Compared with normal liver tissues. **a, b** LPCAT1 protein expression levels are markedly increased in HCC tissues (**c**, **d**) (magnification × 40 and × 400). **e**, **f** Increased LPCAT1 protein expression possessed a strong ability to discriminate between HCC and normal liver tissues (P < 0.0001). HCC, hepatocellular carcinoma; ****P < 0.0001

As opposed to normal liver tissue specimens (Fig. 2a–b), LPCAT1 protein expression levels were markedly increased in the HCC tissue specimens (Fig. 2c–d), which was consistent with *LPCAT1* mRNA expression. Increased LPCAT1 protein expression possessed a strong ability to discriminate between HCC and normal liver tissues (Fig. 2e–f).

### Poor prognosis of HCC patients with* LPCAT1* upregulation

**Fig. 3:**
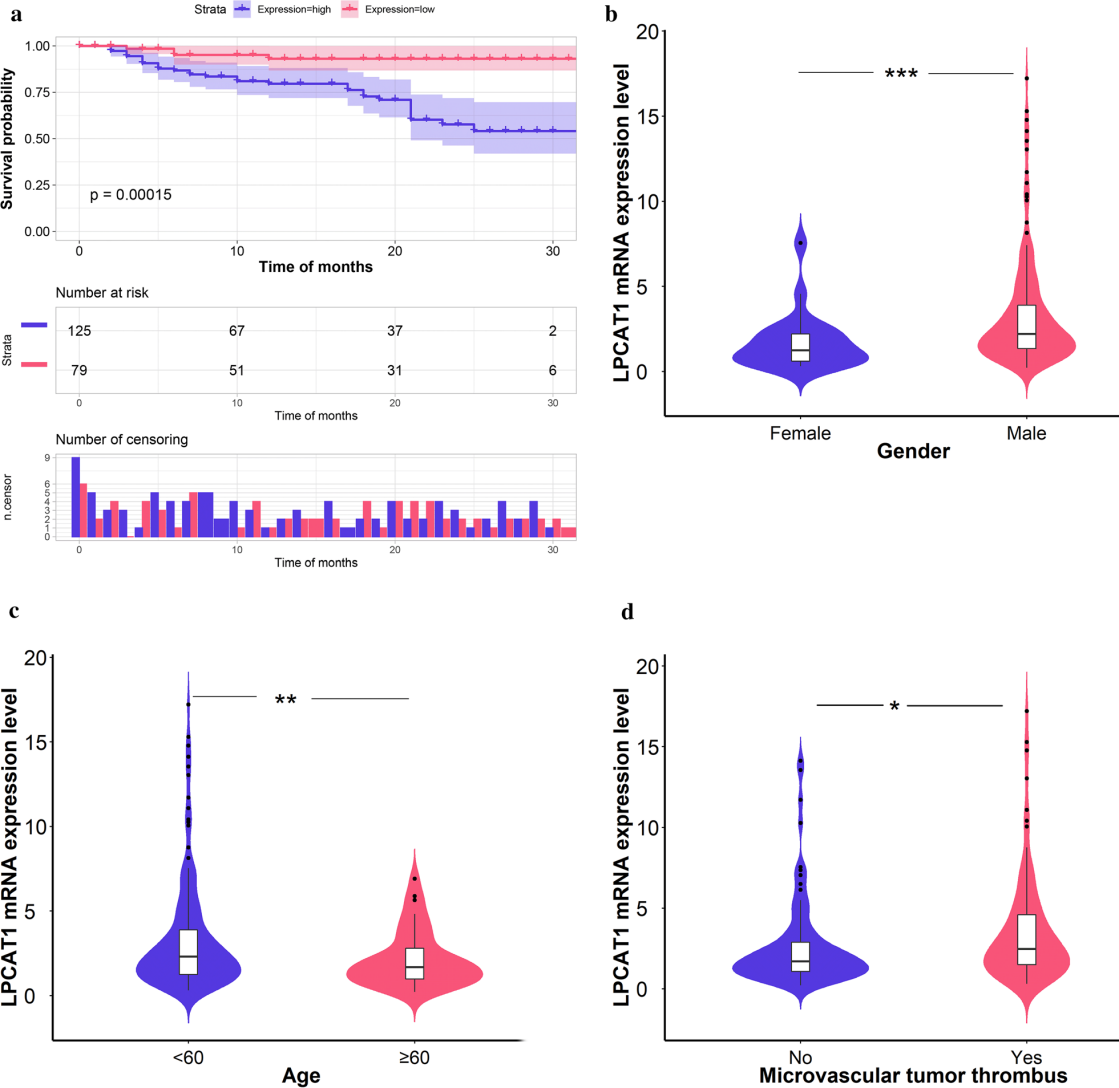
Increased expression of *LPCAT1* is correlated with poor prognosis of HCC patients. **a** Higher *LPCAT1* expression predicts worse overall survival outcomes. **b**–**d** Among HCC patients, elevated expression levels of *LPCAT1* were significantly different in terms of gender, age, and microvascular tumor thrombus. ***P < 0.001; **P < 0.01; *P < 0.05. HCC, hepatocellular carcinoma

The result of Kaplan–Meier survival analysis indicated that higher *LPCAT1* expression forecasted worse overall survival outcomes in HCC patients (Fig. 3a). Increased *LPCAT1* expression patterns varied from gender (male: 3.28 ± 3.10 vs. female: 1.73 ± 1.66; P = 0.001), age (< 60: 3.33 ± 3.22 vs. ≥ 60: 2.17 ± 1.65; P = 0.002) and microvascular tumour thrombus (with thrombus: 3.50 ± 3.20 vs. without thrombus: 2.64 ± 2.71; P = 0.040) (Fig. 3b–d). External HCC cohorts were used to confirm the prognostic value of *LPCAT1* in HCC (Additional file 6: Figure S4a). The GSE10143 cohort was probed to be the primary source of heterogeneity and had been removed (Additional file 6: Figure S4b). Collectively, a combined HR value of 2.21 [1.64, 2.99] indicated that *LPCAT1* might serve as an independent risk factor for HCC (Additional file 6: Figure S4c).

### Establishment of* LPCAT1* knockout HCC cells

**Fig. 4:**
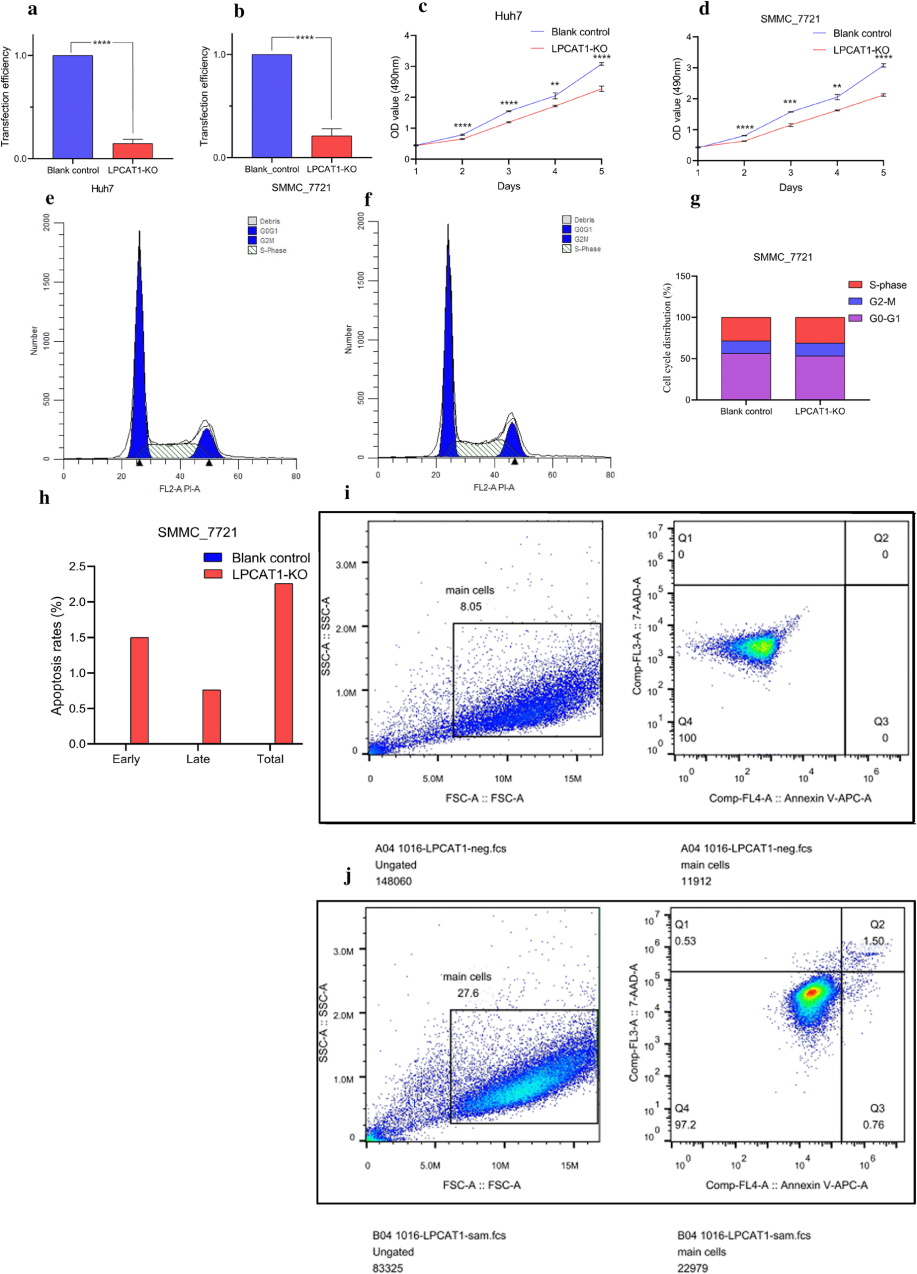
*LPCAT1* induced the proliferation of Huh7 and SMMC-7721 cells. The *LPCAT1*-knockout Huh7 and SMMC-7721 cells were constructed by using the clustered regularly interspaced short palindromic repeats (CRISPR)/associated protein 9 (Cas9) system. **a**, **b** Validated the transfection efficiency of *LPCAT1*-knockout group and control group in Huh7 and SMMC-7721 cell lines, respectively. **c**, **d** Proliferation assay was conducted to evaluate the effect of *LPCAT1*-knockout on cellular proliferation. **e**–**j** Flow cytometry was used to detect the cycle distribution and apoptosis of *LPCAT1*-knockout SMMC-7721 cells. ****P < 0.0001; ***P < 0.001; **P < 0.01

To reveal the cellular biological functions of *LPCAT1*, *LPCAT1* knockout cells were successfully established and were transfected into two HCC-derived cell lines (i.e., SMMC-7721 and Huh7) [20]. The result of the RT-qPCR verification experiment indicated lower *LPCAT1* mRNA expression levels in *LPCAT1* knockout SMMC-7721 cells (P = 4.0E–05) and *LPCAT1* knockout Huh7 cells (P = 3.3E–06) than those in the control group (Fig. 4a–b).

### Inhibited proliferation, migration, and invasion abilities in* LPCAT1* knockout HCC cells

**Fig. 5:**
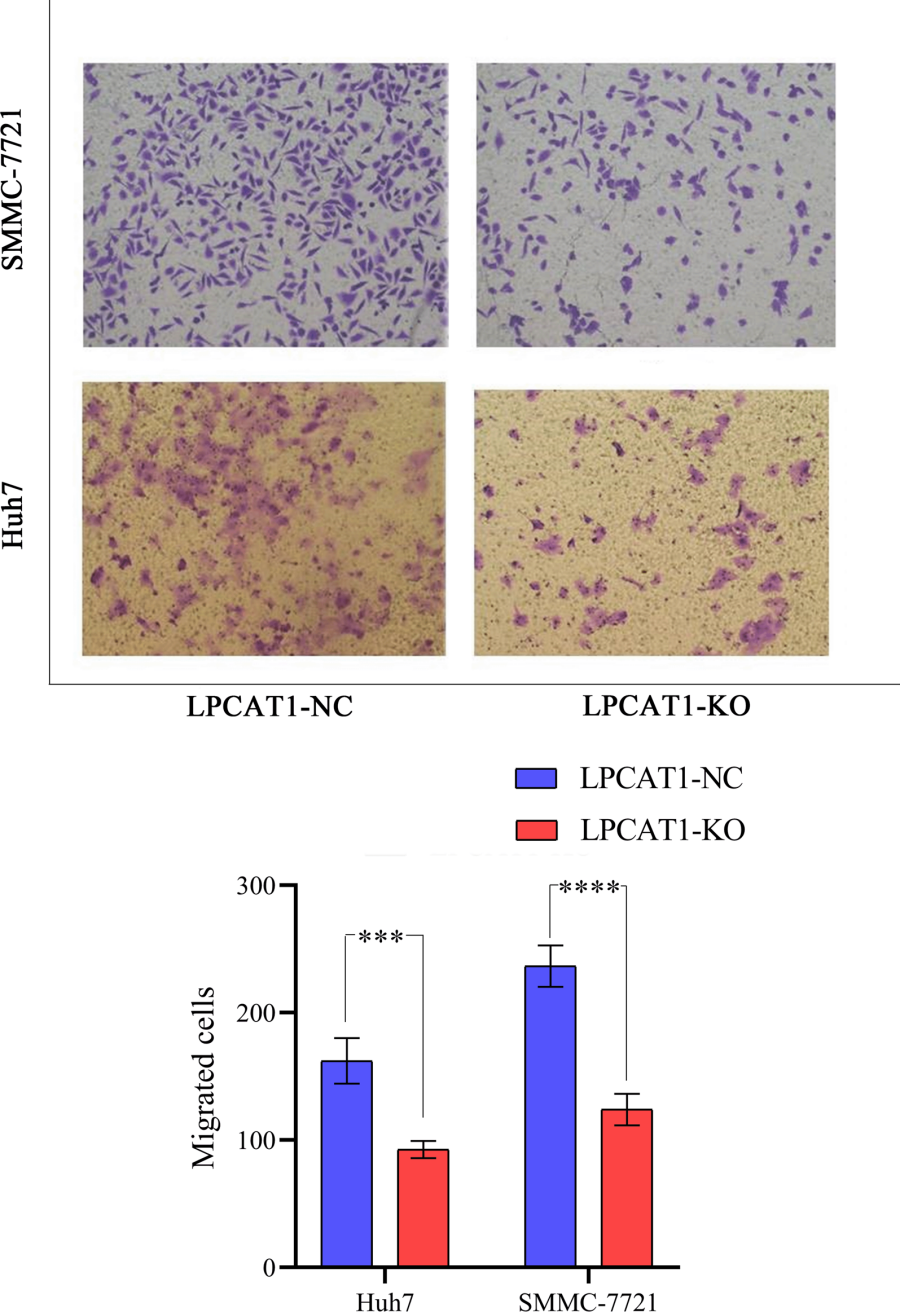
The ability of cell migration to *LPCAT1*-knockout group and control group in SMCC-7721 and Huh7 cell lines. *LPCAT1* might promote the migration of SMCC-7721 and Huh7 cells. ****P < 0.0001; ***P < 0.001

**Fig. 6:**
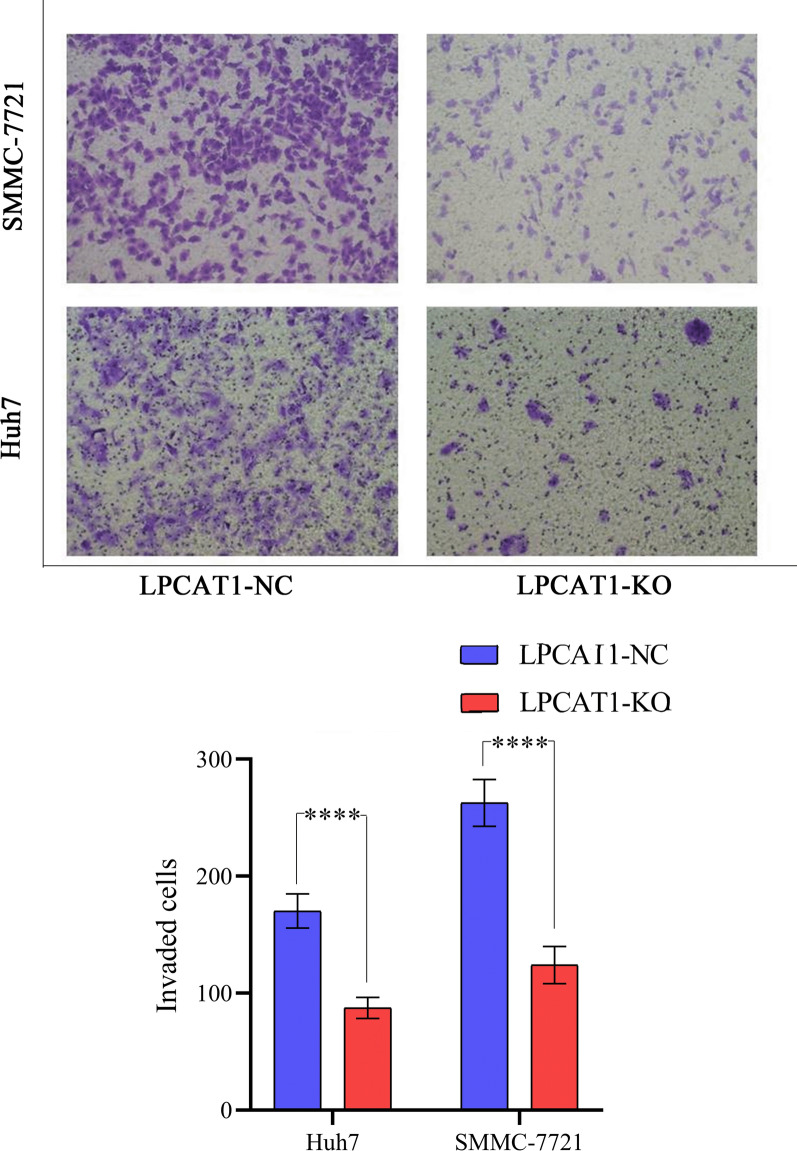
The ability of cell invasion to *LPCAT1*-knockout group and control group in SMCC-7721 and Huh7 cell lines. *LPCAT1* might promote the invasion of SMCC-7721 and Huh7 cells. ****P < 0.0001

It was found that compared with the blank control group, *LPCAT1* knockout significantly hampered cellular growth in SMMC-7721 and Huh7 cells (Fig. 4c–d). Therefore, *LPCAT1* might promote the proliferation of HCC cells. Compared with the *LPCAT1* non-knockout transfected cells in the two cell lines, there was a significant decrease in the number of penetrating *LPCAT1* knockout SMMC-7721 and Huh7 cells (Figs. 5, 6). Therefore, *LPCAT1* might enhance the migration and invasion abilities of SMMC-7721 and Huh7 cells.

### Cell cycle and apoptosis of* LPCAT1* knockout cells

It was observed that compared with the distribution of 56.45% cells in the control group, about 53.32% of *LPCAT1* knockout cells were distributed in the G0/ G1 period. Additionally, about 31.16% of *LPCAT1* knockout cells were distributed in the S period, higher than that of 28.45% cells in the control group (Fig. 4e–g). Therefore, *LPCAT1* knockout might arrest the cell cycle at the S phase. Additionally, the apoptotic rates of *LPCAT1* knockout SMMC-7721 cells slightly increased when compared with the blank control group (Fig. 4h–j). However, the statistical significance cannot be determined because of a lack of duplicate samples.

### Prospective signal transduction pathways of* LPCAT1* in HCC

A total of 5399 HCC DEGs were identified, containing 3736 overexpressed DEGs and 1663 under expressed DEGs, all with significant SMD. Additionally, a total of 1223 *LPCAT1* CEGs were determined, including 564 positive CEGs and 659 negative CEGs. Upregulated DEGs were intersected by positive CEGs, and 473 genes were obtained whose expression level was increased in HCC and were positively correlated with *LPCAT1*. Downregulated DEGs were intersected by negative CEGs, and 367 genes were obtained. Enrichment analysis indicated that the upregulated *LPCAT1* CEGs were dominantly clustered in the cell cycle pathway according to the KEGG (Additional file 7: Figure S5a) and reactome (Additional file 7: Figure S5b). Additionally, embryonal cancer was determined to be the most enriched disease (Additional file 7: Figure S5c). Moreover, mitotic nuclear division, cell-substrate adherens junction, and cadherin binding were found to be the most prominent “Biological Process,” “Cellular Component,” and “Molecular Function,” respectively (Additional file 8: Figure S6). On the other hand, the downregulated *LPCAT1* CEGs were primarily clustered in chemical carcinogenesis (Additional file 9: Figure S7a) and biological oxidations (Additional file 9: Figure S7b). Accordingly, inherited metabolic disorder was found to be the most associated disease (Additional file 9: Figure S7c). Furthermore, small molecule catabolic process, plasma lipoprotein particle, and monooxygenase activity were identified as the most aggregated GO terms according to the downregulated genes negatively related to *LPCAT1* in HCC (Additional file 10: Figure S8). The mutation oncoplot of the cell cycle and chemical carcinogenesis is presented in Additional file 11: Figure S9a–b.

### Construction of the genetic regulatory network of* LPCAT1* in HCC

**Fig. 7:**
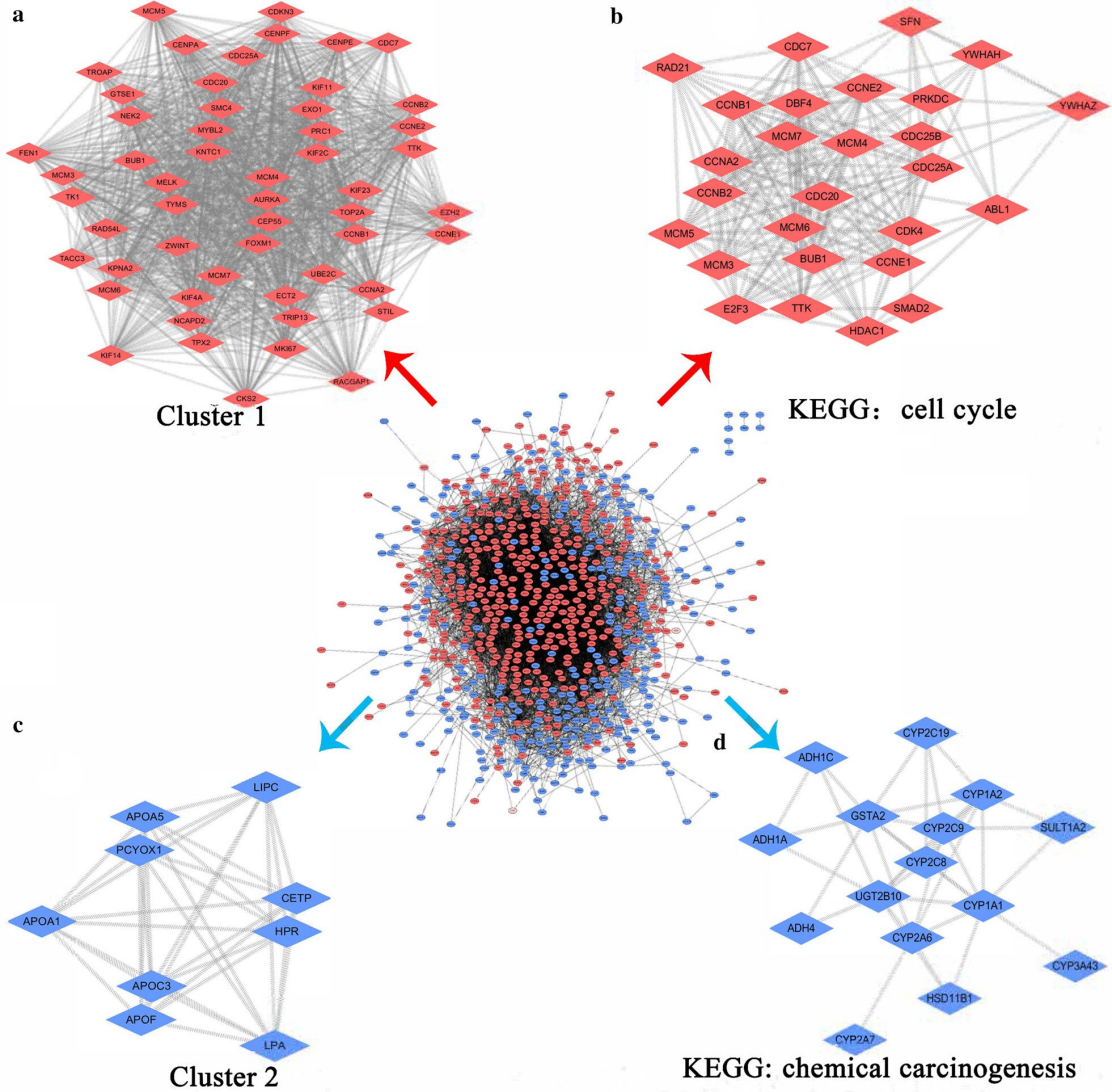
The genetic regulatory network of *LPCAT1*’s differentially co-expressed genes in hepatocellular carcinoma. Gene in blue and red represent downregulation and upregulation, respectively

*LPCAT1*’s genetic regulatory network in HCC was constructed based on the intersected genes that had been described in the “Prospective signal transduction pathways of* LPCAT1* in HCC” section (Fig. 7). Two functional modules (i.e., Cluster 1 and Cluster 2) were identified for the upregulated CEGs and downregulated CEGs, respectively. PPI of the cell cycle and chemical carcinogenesis pathways were explored further. *CCNB1*, which was significantly correlated with *LPCAT1* (Spearman R = 0.58, P = 2E–48), was established as the hub gene in the cell cycle pathway. Moreover, a total of three core genes were established in the chemical carcinogenesis pathway, containing *UGT2B10* (Spearman R = –0.50, P = 3.2E–34), *GSTA2* (Spearman R = –0.30, P = 2.4E–12), and *CYP1A1* (Spearman R = – 0.37, P = 3.1E–18).

### Potential therapeutic agents for HCC by targeting at* LPCAT1*’s regulatory network

By constructing the gene regulatory network based on the DEGs related to *LPCAT1* in HCC, several potential therapeutic agents for HCC were identified by targeting at *LPCAT1*’s regulatory network (Table 1). The molecular formulas of such drugs are presented in Additional file 12: Figure S10. Surprisingly, the anti-HCC effects of LY-294002 [41], trichostatin A [42], sirolimus [43], vorinostat [44], and harmine [45] have been demonstrated by in vitro or in vivo experiments.

**Table 1:** Potential therapeutic agents for hepatocellular carcinoma by targeting *LPCAT1*’s regulatory network

Rank	Cmap name	Cell line	Description	Chemical formula		Enrichment	P		Specificity	Anti-cancer	Anti-HCC
1	LY-294002	MCF7	Specific inhibitor of phosphatidylinositol 3-kinase	C19H17NO3		− 0.437	< 0.0001		0.17	√	√
2	Trichostatin A	PC3	Histone deacetylase 3 inhibitor	C17H22N2O3		− 0.366	< 0.0001		0.53	√	√
3	Trichostatin A	MCF7		C17H22N2O3		− 0.307	< 0.0001		0.67		
4	Sirolimus	MCF7	An immunosuppressant which possesses both antifungal and antineoplastic properties	C51H79NO13		− 0.395	< 0.001		0.36	√	√
5	Tomatidine	MCF7	/	/		− 0.982	< 0.001		0	√	/
6	Pyridoxine	MCF7	The 4-methanol form of vitamin B6	C8H11NO3		− 0.981	< 0.001		0.01	√	/
7	Vorinostat	MCF7	A drug currently under investigation for the treatment of cutaneous T cell lymphoma	C14H20N2O3		− 0.673	< 0.01		0.28	√	√
8	Meticrane	PC3	Diuretic medication	C10H13NO4S2		− 0.976	< 0.01		0	/	/
9	Harmine	MCF7	/	C13H12N2O		− 0.972	< 0.01		0.01	√	√
10	Tanespimycin	MCF7	A small molecule inhibitor of heat shock protein 90 (HSP90), which is developed for the treatment of several types of cancer, solid tumours or chronic myelogenous leukemia	C31H43N3O8		− 0.305	< 0.01		0.49	√	√

## Discussion

Lipid metabolisms have been verified to play profound roles in the onset and deterioration of HCC through lipid metabolic reprogramming and metabolism rearrangements [46–48]. Mounting evidence demonstrated that *LPCAT1* participates in the pathophysiological process of cancer by dysregulating lipid metabolisms [16, 48]. In this study, the authors found that increased *LPCAT1* induced the proliferation, migration, and metastasis of SMMC-7721 and Huh7 cells. A regulatory network of *LPCAT1* was constructed to analyse the molecular mechanisms behind the initiation and progression of HCC. Several small molecule drugs were forecasted by targeting at the network.

There was only one small study that elucidated the upregulation of *LPCAT1* in HCC [22]. By contrast, the present study highlights the biological functions of *LPCAT1* and verifies its upregulated expression patterns in 3715 HCC specimens as opposed to 3105 non-HCC specimens. Furthermore, the prognostic value of *LPCAT1* was comprehensively assessed by in-house and external HCC cohorts. More importantly, the cell cycle and chemical carcinogenesis were identified as the two most enriched signalling pathways. Potential drugs targeting at *LPCAT1* in HCC were predicted.

*LPCAT1* mRNA and protein were abundant in HCC tissues. In-house RT-qPCR, immunohistochemistry, external RNA sequencing, and gene chip were used to confirm the increased expression levels of *LPCAT1* in HCC tissues. A SMD index was used to combine the effect size, which further supported the upregulation of *LPCAT1* (SMD = 0.63 [0.35, 0.90]). Therefore, the results of the present study are more convincing. Additionally, increased *LPCAT1* presages poor prognosis of HCC patients. Higher *LPCAT1* was correlated with worse overall survival conditions in 125 HCC patients, suggesting *LPCAT1* might be a potent risk factor in HCC (combined HR = 2.21 [1.64, 2.99]). Also, *LPCAT1* expression shows an ascending pattern in male, age < 60, and microvascular tumour thrombus HCC patients. Collectively, it is conceivable that upregulated *LPCAT1* might play crucial roles in the unfavourable prognosis of HCC.

*LPCAT1* might promote HCC progression by accelerating cell growth, migration, and metastasis. *LPCAT1* knockout SMMC-7721 and Huh7 cells were established using the CRISPR/Cas9 system [49]. The proliferation, migration, and metastasis abilities of *LPCAT1* knockout transfected SMMC-7721 and Huh7 cells were significantly hampered, thus indicating that *LPCAT1* might participate in the deterioration of HCC cells by promoting the mechanisms of cell growth, migration, and metastasis. This hypothesis can be supported by a previous study, where only one HCC-derived cell line was used [22]. Interestingly, another study reported that *LPCAT1* is required for the miRNA-205-induced proliferation of SMMC-7721 cells [50]. Furthermore, *LPCAT1* knockout might arrest the cell cycle at the S phase and increase the cell apoptotic rates in SMMC-7721 cells, which was reported for the first time. Therefore, the authors speculate that *LPCAT1* might induce the DNA synthesis process and abrogate cell apoptosis to survive HCC cells. However, the influence *LPCAT1* has on the cell cycle distribution and the apoptotic rate of HCC cells has not been verified by duplicate samples. More experimental evidence must be supplemented in the future to support the findings in the present study.

Since *LPCAT1* was important in HCC initiation and progression, the authors sought to shed light on its potential molecular mechanisms underlying HCC. A genetic regulatory network of *LPCAT1* was constructed, where upregulated *LPCAT1* CEGs were prominently clustered in the cell cycle pathway. As is known, aberrant regulation of the cell cycle induces uncontrollable cell proliferation and handles various cancers, including HCC [51–53]. The result of flow cytometry unveils an intimate association between *LPCAT1* and cell cycle regulation; however, the precise regulatory mechanisms had never been studied before. In this study, upregulated *LPCAT1* CEGs were found to participate in mitotic nuclear division, regulation of mitotic cell cycle phase transition, cell cycle checkpoints, and mitotic G1-G1/S phases in terms of GO and reactome. During mitotic nuclear division, the primary event of the M phase, mitotic genes are prone to be activated when the chromatin structures change, which creates favourable conditions for the proliferation of HCC cells [54]. Interestingly, *CCNB1*, the hub gene in the cell cycle pathway, encodes an important regulatory protein in mitosis (cyclin B1). Increased cyclin B1, prominently working as a “switch” in the process of cell cycle, results in malignant proliferation of HCC cells by binding to cyclin-dependent kinases [55]. Furthermore, there was a positive association between the mRNA expression levels of *LPCAT1* and *CCNB1* (Spearman R = 0.58, P = 2E–48), indicating that there might be a synergetic effect between *LPCAT1* and *CCNB1* on the pathogenesis of HCC. Additionally, the cell cycle checkpoint is verified to be one of the most important cell cycle regulatory mechanisms [56]. Nonetheless, dysregulation of the cell cycle checkpoint might lead to radio resistance of HCC cells, thus causing unfavourable prognosis in HCC patients [57]. Therefore, the upregulated *LPCAT1* CEGs might induce abnormal regulation of the cell cycle and promote the development and deterioration of HCC. On the other hand, downregulated *LPCAT1* CEGs provide important clues on metabolism mechanisms, which might propel chemical carcinogenesis. Downregulated *LPCAT1* CEGs are involved in small molecule catabolic processes, plasma lipoprotein particles, and biological oxidations. Surprisingly, increased *LPCAT1* is likely to induce metabolic disturbance and fuel the process of liver damage and carcinogenesis. In liver injury mouse models, *LPCAT1* was upregulated in the carbon tetrachloride-treated mouse, along with increased saturated fatty acylphospholipid in liver tissues and peripheral plasma [58]. Furthermore, another study verified the lipid composition in Huh7 cells by using mass spectrometry imaging [22]. Collectively, it was conceivable that upregulated *LPCAT1* might disturb the metabolism of lipids and promote liver carcinogenesis by co-expressing with downregulated CEGs. More studies are required to further confirm our hypothesis.

Moreover, several therapeutic drugs were successfully predicted by targeting at the genetic regulatory network of *LPCAT1*. As is known, the clinical efficiency of HCC patients was largely limited by chemotherapy resistance and liver dysfunction. It is urgently needed to develop more potent agents with hepatoprotective and anti-HCC properties [59]. In the present study, LY-294002, trichostatin A, sirolimus, vorinostat, and harmine were forecasted to be potential small molecule drugs for treating HCC. LY-294002, an inhibitor of phosphatidylinositol 3-kinase, has proved useful in hampering HCC cell growth, migration, and invasion [60]. Additionally, histone deacetylase inhibitor trichostatin A has seen anti-HCC efficacy by activating cell death signalling cascades [61]. More importantly, a single-centre study demonstrated the favourable effect sirolimus has on HCC patients, whose survival time were longer than in the control group [62]. Therefore, such evidence indicates that repurposing FDA-approved small molecule drugs is a promising direction for HCC treatment. In vivo experiments and clinical trials will promote the applications of these small molecule drugs.

There are several limitations. First, a high level of heterogeneity was detected in the present study. The author used a randomized-effect model to minimize the influence of heterogeneity. In the future, more single cell RNA sequencing experiments will help to reduce intercellular heterogeneity. Second, the influence *LPCAT1* has on the cell cycle distribution and the apoptotic rate of HCC cells has not been verified by different cell lines. Third, the complicated molecular mechanisms and practical effects of the predicted small molecule drugs are unclear. However, the present study sheds light on several novel signalling pathways and reveals potential therapeutic directions for HCC. Further studies are needed to validate the conclusions.

## Conclusions

*LPCAT1* was upregulated and correlated with poor prognosis in HCC patients. Increased *LPCAT1* fuelled HCC progression by promoting cell proliferation, migration, and metastasis. *LPCAT1* co-expressed genes and HCC differentially expressed genes prominently participated in the cell cycle and chemical carcinogenesis pathways.

## Supplementary Information

**Additional file 1**:** Table S1**: Fundamental information of the included datasets.

**Additional file 2**:** Figure S1**: Upregulation of *LPCAT1* in hepatocellular carcinoma based on public datasets.

**Additional file 3**:** Table S2**: Basic statistics of the included datasets.

**Additional file 4**:** Figure S2**: Upregulation of *LPCAT1* in 3715 hepatocellular carcinoma tissues compared with 3105 normal liver tissues. **a** Forest plot of standardized mean differences. **b** Sensitive analysis. **c** Begg’s funnel plot.

**Additional file 5**:** Figure S3**: Potential clinical applications of *LPCAT1* in HCC. *LPCAT1* showed a moderate ability in differentiating HCC tissues from normal liver tissues according to **a** summary receiver operating characteristic curve, **b** forest plot of positive likelihood **c** forest plot of negative likelihood. HCC, hepatocellular carcinoma.

**Additional file 6**:** Figure S4**: Potential prognostic value of *LPCAT1* in HCC. **a** Hazard ratios from in-house and external cohorts of HCC patients were combined. **b** GSE10143 was responsible for a high level of heterogeneity. **c** *LPCAT1* may serve as an independent risk factor for HCC after the removal of GSE10143. HCC, hepatocellular carcinoma.

**Additional file 7**:** Figure S5**: Prospective molecular signaling transduction pathways based on upregulated genes positively related to *LPCAT1* in hepatocellular carcinoma. **a** The Kyoto Encyclopedia of Genes and Genomes pathway. **b** The reactome pathway. **c** The disease ontology.

**Additional file 8**:** Figure S6**: Gene ontology enrichment catalogues based on upregulated genes positively related to *LPCAT1* in hepatocellular carcinoma. Based on the upregulated genes positively related to *LPCAT1* in HCC, mitotic nuclear division, cell-substrate adherens junction, and cadherin binding were found to be the most aggregated gene ontology terms.

**Additional file 9**:** Figure S7**: Prospective molecular signaling transduction pathways based on downregulated genes negatively related to *LPCAT1* in hepatocellular carcinoma. **a** The Kyoto Encyclopedia of Genes and Genomes pathway. **b** The reactome pathway. **c** The disease ontology.

**Additional file 10**:** Figure S8**: Gene ontology enrichment catalogues based on downregulated genes negatively related to *LPCAT1* in hepatocellular carcinoma. Small molecule catabolic process, plasma lipoprotein particle, and monooxygenase activity were identified as the most aggregated gene ontology terms according to the downregulated genes negatively related to *LPCAT1* in hepatocellular carcinoma.

**Additional file 11**:** Figure S9**: Genetic alterations to differentially expressed genes co-expressed with *LPCAT1* in hepatocellular carcinoma. **a** Oncoplot of *LPCAT1* alterations in hepatocellular carcinoma. **b** Oncoplot of differentially expressed genes co-expressed with *LPCAT1* in cell cycle and chemical carcinogenesis pathway.

**Additional file 12**:** Figure S10**: Potential therapeutic agents for hepatocellular carcinoma by targeting *LPCAT1*’s regulatory network. **a** LY-294002 (C19H17NO3). **b** Trichostatin A (C17H22N2O3). **c** Sirolimus (C51H79NO13). **d** Pyridoxine (C8H11NO3). **e** Vorinostat (C14H20N2O3). **f** Harmine (C13H12N2O). **g** Tanespimycin (C31H43N3O8).

## Abbreviations

HCC: Hepatocellular carcinoma
*LPCAT1*: Lysophosphatidylcholine acyltransferase 1
DMEM: Dulbecco’s modified Eagle’s medium
FBS: Fetal bovine serum
IHC: Immunohistochemistry
*ACTB*: Beta-actin
RT-qPCR: Real-time reverse transcription-polymerase chain reaction
*CRISPR/Cas9*: The clustered regularly interspaced short palindromic repeats/associated protein 9
PBS: Phosphate buffered solution
DEGs: Differential expression genes
CEGs: Co-expressed genes
SMD: Standardized mean differences
PCC: Pearson correlation coefficient
GO: Gene ontology
DO: Disease ontology
KEGG: Kyoto Encyclopaedia of Genes and Genomes
STRING: Search tool for the retrieval of interacting genes
PPI: Protein–protein interaction
Cmap: Connectivity map
HR: Hazard ratio
ROC: Receiver operating characteristic curve
SROC: Summary receiver operating characteristic
AUC: Area under the curve

## Declarations

### Availability of data and materials

The datasets analysed during the current study are available in the Gene Expression Omnibus (https://www.ncbi.nlm.nih.gov/geo/), ArrayExpress (https://www.ebi.ac.uk/arrayexpress/), The Genotype-Tissue Expression (https://gtexportal.org/home/), The Cancer Genome Atlas (https://www.cancer.gov/about-nci/organization/ccg/research/structural-genomics/tcga), and Sequence Read Archive (https://www.ncbi.nlm.nih.gov/sra). The accessions of datasets were listed in Additional file 1: Table S1.

## References

[1] Zhao W, Ma B, Tian Z, Han H, Tang J, Dong B (2021). Inhibiting CBX4 efficiently protects hepatocellular carcinoma cells against sorafenib resistance. Br J Cancer.

[2] Fujita M, Yamaguchi R, Hasegawa T, Shimada S, Arihiro K, Hayashi S (2020). Classification of primary liver cancer with immunosuppression mechanisms and correlation with genomic alterations. EBioMedicine.

[3] Siegel RL, Miller KD, Fuchs HE, Jemal A (2021). Cancer statistics, 2021. CA Cancer J Clin.

[4] Zhang H, Dong P, Guo S, Tao C, Chen W, Zhao W (2020). Hypomethylation in HBV integration regions aids non-invasive surveillance to hepatocellular carcinoma by low-pass genome-wide bisulfite sequencing. BMC Med.

[5] Gu X, Guan J, Xu J, Zheng Q, Chen C, Yang Q (2021). Model based on five tumour immune microenvironment-related genes for predicting hepatocellular carcinoma immunotherapy outcomes. J Transl Med.

[6] Liu S, Qiu J, He G, He W, Liu C, Cai D (2021). TRAIL promotes hepatocellular carcinoma apoptosis and inhibits proliferation and migration via interacting with IER3. Cancer Cell Int.

[7] Razavi ZS, Asgarpour K, Mahjoubin-Tehran M, Rasouli S, Khan H, Shahrzad MK (2021). Angiogenesis-related non-coding RNAs and gastrointestinal cancer. Mol Ther Oncol.

[8] Shafabakhsh R, Arianfar F, Vosough M, Mirzaei HR, Mahjoubin-Tehran M, Khanbabaei H, et al. Autophagy and gastrointestinal cancers: the behind the scenes role of long non-coding RNAs in initiation, progression, and treatment resistance. Cancer Gene Ther. 2021. 10.1038/s41417-020-00272-7 33432087

[9] de Martel C, Georges D, Bray F, Ferlay J, Clifford GM (2020). Global burden of cancer attributable to infections in 2018: a worldwide incidence analysis. Lancet Glob Health.

[10] Tian MX, Liu WR, Wang H, Zhou YF, Jin L, Jiang XF (2019). Tissue-infiltrating lymphocytes signature predicts survival in patients with early/intermediate stage hepatocellular carcinoma. BMC Med.

[11] Zhou KQ, Sun YF, Cheng JW, Du M, Ji Y, Wang PX (2020). Effect of surgical margin on recurrence based on preoperative circulating tumor cell status in hepatocellular carcinoma. EBioMedicine.

[12] Zou Y, Li H, Graham ET, Deik AA, Eaton JK, Wang W (2020). Cytochrome P450 oxidoreductase contributes to phospholipid peroxidation in ferroptosis. Nat Chem Biol.

[13] Wang K, Wu Z, Si Y, Tang W, Xu X, Cheng Y (2021). Identification of LPCAT1 expression as a potential prognostic biomarker guiding treatment choice in acute myeloid leukemia. Oncol Lett.

[14] Jiang H, Li Z, Huan C, Jiang XC (2018). Macrophage lysophosphatidylcholine acyltransferase 3 deficiency-mediated inflammation is not sufficient to induce atherosclerosis in a mouse model. Front Cardiovasc Med.

[15] Wang B, Tontonoz P (2019). Phospholipid remodeling in physiology and disease. Annu Rev Physiol.

[16] Lebok P, von Hassel A, Meiners J, Hube-Magg C, Simon R, Höflmayer D (2019). Up-regulation of lysophosphatidylcholine acyltransferase 1 (LPCAT1) is linked to poor prognosis in breast cancer. Aging.

[17] Du Y, Wang Q, Zhang X, Wang X, Qin C, Sheng Z (2017). Lysophosphatidylcholine acyltransferase 1 upregulation and concomitant phospholipid alterations in clear cell renal cell carcinoma. J Exp Clin Cancer Res CR.

[18] Mansilla F, da Costa KA, Wang S, Kruhøffer M, Lewin TM, Orntoft TF (2009). Lysophosphatidylcholine acyltransferase 1 (LPCAT1) overexpression in human colorectal cancer. J Mol Med (Berl).

[19] Wei C, Dong X, Lu H, Tong F, Chen L, Zhang R (2019). LPCAT1 promotes brain metastasis of lung adenocarcinoma by up-regulating PI3K/AKT/MYC pathway. J Exp Clin Cancer Res CR.

[20] Shida-Sakazume T, Endo-Sakamoto Y, Unozawa M, Fukumoto C, Shimada K, Kasamatsu A (2015). Lysophosphatidylcholine acyltransferase1 overexpression promotes oral squamous cell carcinoma progression via enhanced biosynthesis of platelet-activating factor. PLoS ONE.

[21] Han C, Yu G, Mao Y, Song S, Li L, Zhou L (2020). LPCAT1 enhances castration resistant prostate cancer progression via increased mRNA synthesis and PAF production. PLoS ONE.

[22] Morita Y, Sakaguchi T, Ikegami K, Goto-Inoue N, Hayasaka T, Hang VT (2013). Lysophosphatidylcholine acyltransferase 1 altered phospholipid composition and regulated hepatoma progression. J Hepatol.

[23] Bi J, Ichu TA, Zanca C, Yang H, Zhang W, Gu Y (2019). Oncogene amplification in growth factor signaling pathways renders cancers dependent on membrane lipid remodeling. Cell Metab.

[24] Han Y, Zhuang Q, Sun B, Lv W, Wang S, Xiao Q (2021). Crystal structure of steroid reductase SRD5A reveals conserved steroid reduction mechanism. Nat Commun.

[25] Bidar M, Bahlakeh A, Mahmoudi M, Ahrari F, Shahmohammadi R, Jafarzadeh H. Does the application of GaAlAs laser and platelet-rich plasma induce cell proliferation and increase alkaline phosphatase activity in human dental pulp stem cells? Lasers in medical science. 2021. 10.1007/s10103-020-03239-0 33459924

[26] Wu HY, Cai KT, Ma J, Chen G, Dang YW, Lu HP (2019). Evaluation of miR-302b-5p expression and molecular mechanism in hepatocellular carcinoma: findings based on RT-qPCR and in silico analysis. Pathol Res Pract.

[27] Xie Z, Dang Y, Wu H, He R, Ma J, Peng Z (2020). Effect of CELSR3 on the cell cycle and apoptosis of hepatocellular carcinoma cells. J Cancer.

[28] Gao L, Yan SB, Yang J, Kong JL, Shi K, Ma FC (2020). MiR-182-5p and its target HOXA9 in non-small cell lung cancer: a clinical and in-silico exploration with the combination of RT-qPCR, miRNA-seq and miRNA-chip. BMC Med Genomics.

[29] Gao L, Xiong DD, He RQ, Yang X, Lai ZF, Liu LM (2019). MIR22HG as a tumor suppressive lncRNA in HCC: a comprehensive analysis integrating RT-qPCR, mRNA-Seq, and microarrays. OncoTargets Ther.

[30] Itkonen HM, Urbanucci A, Martin SE, Khan A, Mathelier A, Thiede B (2019). High OGT activity is essential for MYC-driven proliferation of prostate cancer cells. Theranostics.

[31] Zhou C, Liu C, Liu W, Chen W, Yin Y, Li CW (2020). SLFN11 inhibits hepatocellular carcinoma tumorigenesis and metastasis by targeting RPS4X via mTOR pathway. Theranostics.

[32] Nambiar DK, Aguilera T, Cao H, Kwok S, Kong C, Bloomstein J (2019). Galectin-1-driven T cell exclusion in the tumor endothelium promotes immunotherapy resistance. J Clin Invest.

[33] Shan D, Zou L, Liu X, Shen Y, Cai Y, Zhang J (2020). Efficacy and safety of gabapentin and pregabalin in patients with vasomotor symptoms: a systematic review and meta-analysis. Am J Obstet Gynecol.

[34] Zhang L, Luo B, Dang YW, He RQ, Chen G, Peng ZG (2019). The clinical significance of endothelin receptor type B in hepatocellular carcinoma and its potential molecular mechanism. Exp Mol Pathol.

[35] Hong W, Yuan H, Gu Y, Liu M, Ji Y, Huang Z (2020). Immune-related prognosis biomarkers associated with osteosarcoma microenvironment. Cancer Cell Int.

[36] Bi F, Chen Y, Yang Q (2020). Significance of tumor mutation burden combined with immune infiltrates in the progression and prognosis of ovarian cancer. Cancer Cell Int.

[37] Kwon OS, Lee H, Kong HJ, Kwon EJ, Park JE, Lee W (2020). Connectivity map-based drug repositioning of bortezomib to reverse the metastatic effect of GALNT14 in lung cancer. Oncogene.

[38] Zhang C, Chen T, Li Z, Liu A, Xu Y, Gao Y, et al. Depiction of tumor stemlike features and underlying relationships with hazard immune infiltrations based on large prostate cancer cohorts. Briefings in bioinformatics. 2020. 10.1093/bib/bbaa211 32856039

[39] Wang Y, Kong W, Wang L, Zhang T, Huang B, Meng J (2020). Multiple-purpose connectivity map analysis reveals the benefits of esculetin to hyperuricemia and renal fibrosis. Int J Mol Sci.

[40] Liu Z, Wang X, Yang G, Zhong C, Zhang R, Ye J (2020). Construction of lncRNA-associated ceRNA networks to identify prognostic lncRNA biomarkers for glioblastoma. J Cell Biochem.

[41] Ye G, Qin Y, Wang S, Pan D, Xu S, Wu C (2019). Lamc1 promotes the Warburg effect in hepatocellular carcinoma cells by regulating PKM2 expression through AKT pathway. Cancer Biol Ther.

[42] Li YL, Rao MJ, Zhang NY, Wu LW, Lin NM, Zhang C (2019). BAY 87-2243 sensitizes hepatocellular carcinoma Hep3B cells to histone deacetylase inhibitors treatment via GSK-3β activation. Exp Ther Med.

[43] Ferrín G, Guerrero M, Amado V, Rodríguez-Perálvarez M, De la Mata M (2020). Activation of mTOR signaling pathway in hepatocellular carcinoma. Int J Mol Sci.

[44] Li YL, Zhang NY, Hu X, Chen JL, Rao MJ, Wu LW (2018). Evodiamine induces apoptosis and promotes hepatocellular carcinoma cell death induced by vorinostat via downregulating HIF-1α under hypoxia. Biochem Biophys Res Commun.

[45] Miao JF, Peng YF, Chen S, Gao WJ, Yang QX, Zhu P (2018). A novel harmine derivative, *N*-(4-(hydroxycarbamoyl)benzyl)-1-(4-methoxyphenyl)-9*H*-pyrido[3,4-b]indole-3-carboxamide (HBC), as histone deacetylase inhibitor: in vitro antiproliferation, apoptosis induction, cell cycle arrest, and antimetastatic effects. Eur J Pharmacol.

[46] Berndt N, Eckstein J, Heucke N, Gajowski R, Stockmann M, Meierhofer D (2019). Characterization of lipid and lipid droplet metabolism in human HCC. Cells.

[47] Sangineto M, Villani R, Cavallone F, Romano A, Loizzi D, Serviddio G (2020). Lipid metabolism in development and progression of hepatocellular carcinoma. Cancers (Basel)..

[48] Tian Y, Yang B, Qiu W, Hao Y, Zhang Z, Yang B (2019). ER-residential Nogo-B accelerates NAFLD-associated HCC mediated by metabolic reprogramming of oxLDL lipophagy. Nat Commun.

[49] Zhan T, Rindtorff N, Betge J, Ebert MP, Boutros M (2019). CRISPR/Cas9 for cancer research and therapy. Semin Cancer Biol.

[50] Liu F, Wu Y, Liu J, Ni RJ, Yang AG, Bian K (2020). A miR-205-LPCAT1 axis contributes to proliferation and progression in multiple cancers. Biochem Biophys Res Commun.

[51] Zhao Y, Zhu C, Chang Q, Peng P, Yang J, Liu C (2020). MiR-424-5p regulates cell cycle and inhibits proliferation of hepatocellular carcinoma cells by targeting E2F7. PLoS ONE.

[52] Zhu M, Xu W, Wei C, Huang J, Xu J, Zhang Y (2019). CCL14 serves as a novel prognostic factor and tumor suppressor of HCC by modulating cell cycle and promoting apoptosis. Cell Death Dis.

[53] Ying H, Ji L, Xu Z, Fan X, Tong Y, Liu H (2020). TRIM59 promotes tumor growth in hepatocellular carcinoma and regulates the cell cycle by degradation of protein phosphatase 1B. Cancer Lett.

[54] Tian L, Yao K, Liu K, Han B, Dong H, Zhao W (2020). PLK1/NF-κB feedforward circuit antagonizes the mono-ADP-ribosyltransferase activity of PARP10 and facilitates HCC progression. Oncogene.

[55] Xie B, Wang S, Jiang N, Li JJ (2019). Cyclin B1/CDK1-regulated mitochondrial bioenergetics in cell cycle progression and tumor resistance. Cancer Lett.

[56] Willis L, Jönsson H, Huang KC (2020). Limits and constraints on mechanisms of cell-cycle regulation imposed by cell size-homeostasis measurements. Cell Rep.

[57] Sun J, Zhu Z, Li W, Shen M, Cao C, Sun Q (2020). UBE2T-regulated H2AX monoubiquitination induces hepatocellular carcinoma radioresistance by facilitating CHK1 activation. J Exp Clin Cancer Res CR.

[58] Jia M, Peng Z, Yang K, Su C, Wang Y, Yan C (2020). A high-throughput targeted metabolomics method for the quantification of 104 non-polar metabolites in cholesterol, eicosanoid, and phospholipid metabolism: application in the study of a CCl(4)-induced liver injury mouse model. Analyst.

[59] Sarvizadeh M, Hasanpour O, Naderi Ghale-Noie Z, Mollazadeh S, Rezaei M, Pourghadamyari H (2021). Allicin and digestive system cancers: from chemical structure to its therapeutic opportunities. Front Oncol.

[60] Zhang X, Shao J, Li X, Cui L, Tan Z (2019). Docetaxel promotes cell apoptosis and decreases SOX2 expression in CD133-expressing hepatocellular carcinoma stem cells by suppressing the PI3K/AKT signaling pathway. Oncol Rep.

[61] Tsilimigras DI, Ntanasis-Stathopoulos I, Moris D, Spartalis E, Pawlik TM (2018). Histone deacetylase inhibitors in hepatocellular carcinoma: a therapeutic perspective. Surg Oncol.

[62] Zhou L, Pan LC, Zheng YG, Du GS, Fu XQ, Zhu ZD (2018). Novel strategy of sirolimus plus thymalfasin and huaier granule on tumor recurrence of hepatocellular carcinoma beyond the UCSF criteria following liver transplantation: a single center experience. Oncol Lett.

