# Supplementary material for: LPCAT1 overexpression promotes the progression of hepatocellular carcinoma
Source: Cancer Cell Int. 2021 Aug 21;21:442. doi: 10.1186/s12935-021-02130-4 (PMC8380368; doi:10.1186/s12935-021-02130-4)
Supplement: Supplementary file 1 — Additional file 1: Table S1: Fundamental information of the included datasets. [file 12935_2021_2130_MOESM1_ESM.docx]

**Additional file 1 Table S1**: Fundamental information of the included datasets.

| **Dataset** |  | **HCC** |  |  | **Control** |  | **Accession** |
| --- | --- | --- | --- | --- | --- | --- | --- |
|  | **N** | **M** | **SD** | **N** | **M** | **SD** |  |
| In-house  RT-qPCR | 204 | 3.11 | 3.01 | 204 | 1.94 | 1.40 | / |
| GSE10143 | 80 | 12.81 | 0.89 | 307 | 11.88 | 0.68 | GSE10143 |
| GSE124535 | 35 | 2.73 | 1.01 | 35 | 2.02 | 0.41 | GSE124535 |
| GSE22058-GPL6793 | 100 | 10.73 | 1.08 | 97 | 10.28 | 0.46 | GSE22058 |
| GSE54238 | 26 | 10.61 | 1.17 | 30 | 9.42 | 1.25 | GSE54238 |
| GSE59259 | 8 | 10.22 | 0.92 | 8 | 9.45 | 0.34 | GSE59259 |
| GSE74656 | 5 | 8.28 | 0.41 | 5 | 7.06 | 0.35 | GSE74656 |
| Affymetrix | 1583 | 4.20 | 0.59 | 1241 | 4.15 | 0.39 | GSE101685/GSE102079/GSE107170/GSE112790/GSE121248/GSE12941/GSE14323/ |
|  | | | | | | | GSE14520-GPL3921/GSE14520-GPL571/GSE17548/GSE17967/GSE19665/GSE29721/ |
|  |  |  |  |  |  |  | GSE33006/GSE41804/GSE45050/GSE45436/GSE60502/GSE6222/GSE62232/GSE63898/ |
|  |  |  |  |  |  |  | GSE64041/GSE6764/GSE84005/GSE84402/GSE9839 |
| Agilent | 186 | 3.10 | 0.40 | 131 | 3.19 | 0.30 | GSE101728/GSE115018/GSE117361/GSE46408/GSE50579/GSE54236/GSE57555-GPL16699/ GSE67764/GSE98269-GPL21047 |
| Illumina | 1117 | 5.40 | 0.56 | 822 | 5.04 | 0.44 | GSE104310/GSE112221/GSE20140-GPL18461/GSE25599/GSE31370/GSE33294/  GSE36376/GSE36411/GSE39791/ GSE46444/GSE55048/GSE56545/GSE57727/ |
|  | | | | | | | GSE57957/GSE63018/ GSE63863/GSE65485/GSE69164/GSE73708/ GSE76427/ |
|  |  |  |  |  |  |  | GSE77314/GSE77509/GSE81550/ GSE87630/GSE89377/GSE94660/GSE97214/GSE98617 |
| TCGA-GTEx | 371 | 10.44 | 1.38 | 225 | 9.70 | 0.99 | TCGA/GTEx |
| **Total** | **3715** |  |  | **3105** |  |  |  |
