# Supplementary material for: LPCAT1 overexpression promotes the progression of hepatocellular carcinoma
Source: Cancer Cell Int. 2021 Aug 21;21:442. doi: 10.1186/s12935-021-02130-4 (PMC8380368; doi:10.1186/s12935-021-02130-4)
Supplement: Supplementary file 3 — Additional file 3: Table S2: Basic statistics of the included datasets. [file 12935_2021_2130_MOESM3_ESM.docx]

**Additional file 3 Table S2**: Basic statistics of the included datasets. AUC, area under the curve; TP, true positive rate; FP, false positive rate; FN, false negative rate; TN, true negative rate.

| **Dataset** | **AUC** | **TP** | **FP** | **FN** | **TN** |
| --- | --- | --- | --- | --- | --- |
| In-house RT-qPCR | 0.62 | 126 | 86 | 78 | 118 |
| GSE10143 | 0.84 | 63 | 60 | 17 | 247 |
| GSE124535 | 0.76 | 19 | 1 | 16 | 34 |
| GSE22058-GPL6793 | 0.65 | 36 | 1 | 64 | 96 |
| GSE54238 | 0.78 | 24 | 11 | 2 | 19 |
| GSE59259 | 0.84 | 7 | 1 | 1 | 7 |
| GSE74656 | 1 | 5 | 0 | 0 | 5 |
| Affymetrix | 0.5 | 318 | 71 | 1265 | 1170 |
| Agilent | 0.56 | 61 | 19 | 125 | 112 |
| Illumina | 0.69 | 551 | 146 | 566 | 676 |
| TCGA-GTEx | 0.67 | 224 | 72 | 147 | 153 |
